# *Helicobacter pylori* and Hepatitis C Virus Coinfection in Egyptian Patients

**DOI:** 10.4103/0974-777X.59244

**Published:** 2010

**Authors:** Samir El-Masry, Mohamed El-Shahat, Gamal Badra, Mohamed F Aboel-Nour, Mahmoud Lotfy

**Affiliations:** 1 *Department of Molecular Biology, Genetic Engineering and Biotechnology Research Institute, Minufiya University, Sadat City, Minufiya, Egypt, Saudi Arabia*; 2 *Department of Hepatology, National Liver Institute, Minufiya University, Minufiya, Egypt, Saudi Arabia*; 3 *Department of Zoology, Faculty of Science, Mansoura University, Mansoura, Egypt, Saudi Arabia*; 4 *Department of Applied Medical Sciences, Jouf University, Qurayat, Saudi Arabia*

**Keywords:** Child-Pugh, Hepatitis C virus, *Helicobacter pylori*, Meld

## Abstract

**Introduction:**

Chronic hepatitis C virus (HCV) infection is a leading cause of end-stage liver disease worldwide. It has been shown that *Helicobacter pylori* (*H. pylori*) plays an important role in chronic gastritis, peptic ulcer disease and gastric malignancies, and its eradication has been advocated. The association between *H. pylori* infection and liver cirrhosis in patients with hepatitis C virus has been documented in different parts of the world; nevertheless, no conclusive data is available in Egypt.

**Materials and Methods:**

In the present study, the status of *H. pylori* infection was sought in 90 patients with chronic HCV infection and in 66 HCV-free healthy controls.

**Results:**

The study showed that the *H. pylori* positivity was increased significantly (*P* = 0.03) in the HCV-infected patients when compared to that in healthy controls, where *H. pylori* infection was found in 50 (55.6%) out of 90 of the HCV-infected patients versus 26 (39.4%) out of 66 of the healthy controls. In HCV-infected patients, the prevalence of *H. pylori* infection was increased significantly (*P* = 0.04) from chronic active hepatitis to cirrhosis. *H. pylori* infection was present in 6/18 (33.3%), 10/21 (47.6%), 16/27 (59.3%), 18/24 (75.0%) patients with chronic active hepatitis, Child-Pugh score A, Child-Pugh score B and Child-Pugh score C, respectively. More importantly, the prevalence of *H. pylori* infection in HCV-infected patients was increased very significantly (*P* = 0.003) with increasing Meld (model for end-stage liver disease) score. The prevalence of *H. pylori* was documented in 9/28 (32.1%) patients with Meld score >10 and in 41/62 (66.1%) patients with Meld score >10.

**Conclusion:**

It may be stated that our results collectively reflect a remarkable increase in *H. pylori* prevalence with advancing hepatic lesions, and the eradication treatment may prove beneficial in those patients with chronic hepatitis C.

## INTRODUCTION

Hepatitis C virus (HCV) is the leading cause of chronic liver disease globally[1] and is estimated to infect about 170 million people around the world.[2] Chronic HCV infection frequently leads to liver fibrosis and cirrhosis and is associated with the occurrence of hepatocellular carcinoma.[3]

*H. pylori* is recognized as a pathogen of upper gastrointestinal diseases, such as acute and chronic gastritis, duodenal and gastric ulcers[4,5] and mucosa-associated lymphoid tissue (MALT) lymphoma.[6] *H. pylori* has also been closely associated with development of gastric adenocarcinoma.[7] *H. pylori* has been reported to induce hepatotoxicity *in vitro.*[8] A soluble factor that exhibits cytotoxic effects on a mouse liver cell line was identified in the culture medium of *H. pylori* and other Helicobacter species. Furthermore, several investigators have reported a high prevalence of *H. pylori* infection in patients with chronic liver diseases.[9,10] Although *H. pylori* is generally believed to be sensitive to bile,[11–13] several studies have shown that *H. pylori* is detectable in the liver and biliary tract[14–17] and that *H. pylori* can survive in bile-rich environment.[18–20] These findings indicate that bile-resistant *H. pylori* may survive in the liver and biliary tract.

The association between *H. pylori* infection and cirrhosis in patients with hepatitis C virus has been documented in different parts of the world; nevertheless, no conclusive data is available in Egypt. Therefore, the rationale of the present study was to investigate the status of *H. pylori* infection in HCV-infected patients with and without liver cirrhosis.

## MATERIALS AND METHODS

### Study subjects

The present study was conducted on 90 patients from among the out-clinic patients of National Liver Institute (NLI), Minufiya University, Minufiya, Egypt. Sixty-six healthy controls were recruited from among the blood donor clientele of the NLI for comparison purposes. All patients and controls were subjected to thorough history-taking; complete clinical examination; abdominal ultrasound; and laboratory investigations, including total bilirubin (TB), direct bilirubin (DB), total protein (TP), serum albumin (S. Alb), aspartate aminotransferase (AST), alanine aminotransferase (ALT), alkaline phosphatase (ALP) and serum creatinine. Hepatitis B surface antigen (HBsAg), anti-HCV antibodies were detected by ELISA (Diasorium kit; Diasorium SR, Italy) and RT-PCR for HCV RNA (Amplicor PCR; Roche Molecular Systems, Inc., Pleasanton, Calif., USA). All patients were strictly positive for anti-HCV and HCV RNA and negative for HBV. On the other hand, subjects of the control group were free of both HCV and HBV.

Liver biopsy was performed for the patient groups only. Two pathologists did the histopathological assessment separately, and then a consensus between them was made on discordant assessments. The two pathologists were not aware of the clinical data, at the time of assessment. Histological grading of hepatic cirrhosis was done as defined by Ishak *et al.*[21] According to the results of abdominal ultrasonography and liver biopsy, the patients were classified into four groups. The first group comprised 18 patients with chronic active hepatitis, the second group included 21 patients with liver cirrhosis Child-Pugh score A, the third group comprised 27 patients with liver cirrhosis Child-Pugh score B and the last group included 24 patients with liver cirrhosis Child-Pugh score C.

The study protocol respected the most recent Declaration of Helsinki,[22] and all the patients gave consent to the use of their sera and clinical data for research purposes after being informed about the nature of the study.

### Assessment of severity of liver disease using Child and Meld scores

Assessment of severity of liver disease was performed using both Child-Pugh scoring system[23] and the Meld (model for end-stage liver disease) score; the latter was calculated according to the original formula proposed by Mayo Clinic group:

Meld score = 0.957 × loge (creatinine mg/dL) + 0.378 × loge (bilirubin mg/dL) + 1.120 × loge (INR) + 0.643. The Meld score can be easily calculated using a web site. The web site that we have used for our calculations is http://depts.washington.edu/uwhep/calculations/meldscore.htm. The Meld score stratified the patients into two categories — the first group with Meld score ≤10; and the second group, >10.

### Detection of *H. pylori infection*

Gastric mucosal biopsies were used for routine rapid urease testing. The patients were tested for serum *H. pylori* antibodies (Whittaker Bioproducts Inc., Walkersville, MD). A positive result in either of the tests was considered as indicative of active *H. pylori* infection as proved by gastric biopsy positivity. Methodology described by us earlier was followed.[24]

### Statistical analysis

Quantitative data were expressed as mean ± standard deviation. Comparing of two groups was analyzed by Mann-Whitney *U* test, while Kurskal-Wallis test was performed to compare more than two groups. Nominal data were analyzed using chi-square test, and *P* values <0.05 were considered statistically significant. Data were tabulated and analyzed using the SPSS 11 statistical package (SPSS Inc., Chicago, IL).

## RESULTS

### Prevalence of *H. pylori* in HCV-infected patients with different Child and Meld scores

Table 1 summarizes the characteristics of both the patient group and the control group. The results showed that *H. pylori* positivity was increased significantly (*P* = 0.03) in the HCV-infected patients when compared to that of healthy controls, where *H. pylori* infection was found in 50 (55.6%) out of 90 of the HCV-infected patients versus 26 (39.4%) out of 66 of the healthy controls. In HCV-infected patients, the prevalence of *H. pylori* infection was increased significantly (*P* = 0.04) from chronic active hepatitis to cirrhosis. *H. pylori* was present in 6/18 (33.3%), 10/21 (47.6%), 16/27 (59.3%), 18/24 (75.0%) patients with chronic active hepatitis, Child-Pugh score A, Child-Pugh score B and Child-Pugh score C, respectively. More importantly, the prevalence of *H. pylori* infection in HCV-infected patients was increased very significantly (*P* = 0.003) with increasing Meld score. The prevalence of *H. pylori* was documented in 9/28 (32.1%) patients with Meld score ≤10 and in 41/62 (66.1%) patients with Meld score >10 [Table 2].

**Table 1:** Characteristics of the patients and the control groups

Variable	Patients		Control	
		
	No	%	No.	%
*H. pylori*				
Negative	40	44.4	40	60.6
Positive	50	55.6	26	39.4
Schistosoma				
Negative	32	35.6	66	100
Positive	58	64.4	0	0
Sex			
Male	58	64.4	31	47
Female	32	35.6	35	53
Age				
< 40 yrs	29	32	38	57.6
≥ 40 yrs	61	68	28	42.4
Total	90	100	66	100

**Table 2:** Relationship of *H. pylori* to different pathologic, biochemical and demographic characteristics in HCV infected patients

Variable	Positive *H. pylori*		Negative *H. pylori*		No	Total	*P* value
					
	No	%	No	%			
Sex							
Male	37	63.8	21	36.2	58		0.029
Female	13	40.6	19	59.4	32	90	
Age								
< 40 yrs	13	44.8	16	55.2	29		0.11
≥ 40 yrs	37	60.7	24	39.3	61	90	
ALT								
Normal	32	54.2	27	45.8	59		0.4
Elevated	18	58.1	13	41.9	31	90	
AST								
Normal	10	41.7	14	85.3	24		0.08
Elevated	40	60.6	26	39.4	66	90	
S. Bilirubin								
Normal	7	41.2	10	58.8	17		0.14
Elevated	43	58.9	30	41.1	13	90	
Meld score								
≤ 10	9	32.1	19	69.7	28		0.003
> 10	41	66.1	21	33.9	62	90	
Child score								
CAH	6	33.3	12	36.2	18		0.04
A	10	47.6	11	52.4	21		
B	16	59.3	11	40.7	27		
C	18	75.0	6	25.0	24	90	
Schisto.								
Negative	12	37.5	20	62.5	32		0.01
Positive	37	65.5	20	34.5	58	90	

Schisto: Schistosoma infection; CAH: Chronic active hepatitis

### Relation of *H. pylori* with biochemical and demographic characteristics of HCV patients

There were significant differences (*P* = 0.0001 for each) in the mean values of ALT, AST, prothrombin concentration, serum total bilirubin, serum albumin and platelet count between chronic hepatitis C patients and controls. In HCV patients, the number of patients with elevated ALT, AST and total bilirubin was increased in the *H. pylori*–positive group of patients when compared with the *H. pylori*–negative group despite no significance being observed. In the HCV-infected patients, the *H. pylori*–positive patients were likely to be older in age, but there was no significant difference (*P* = 0.11). Also, there were more *H. pylori*–positive male patients than female patients (*P* = 0.029). In the HCV-infected patients, the concurrent schistosoma infection was documented largely in 38/50 (76%) *H. pylori*–positive patients versus 12/50 (24%) patients that were free of schistosoma infection (*P* = 0.010) [Table 2].

It seems clear that sex being male, age being above 40 years and positive schistosoma infection are risk factors for increase in the severity of liver disease, as revealed by Meld score in *H. pylori*–positive patients [Table 3].

**Table 3:** Severity of liver disease as revealed with MELD score in *H. pylori* infected patients in relation to sex, age and schistosoma infection

Patients	MELD		*H. pylori*		No.	Total	*P value*
				
	Positive	%	Negative	%			
Sex male								
≤ 10	7	36.8	12	63.2	19	58	0.004
> 10	30	76.9	9	23.1	39		
Sex female								
≤ 10	2	22.2	7	77.8	9	32	0.17
> 10	11	47.8	12	52.2	23	90	
Age < 40 yrs								
≤ 10	5	35.7	9	64.3	14	29	0.28
> 10	8	53.3	7	46.7	15		
Age ≥ 40 yrs								
≤ 10	4	28.6	10	71.4	14	61	0.007
> 10	33	70.2	14	29.8	47	90	
Schistosoma (−ve)								
≤ 10	3	20	12	80	15	32	0.09
> 10	9	52.9	8	47.1	17		
Schistosoma (+ve)								
≤ 10	6	46.2	7	53.2	13	58	0.05
> 10	32	71.1	13	28.9	45	90	
Total	50		40		90		

## DISCUSSION

Approximately 50% of the humanity is infected with *H. pylori.*[25] *H. pylori* infection in our HCV patients was found to increase non significantly with age and significantly with sex being male. These results are comparable with the data reported by Pellicano *et al.*[10] The prevalence of *H. pylori* infection increases with age but is quite different among the various populations. In United States, the prevalence is less than 20% at 20 years of age, and then increases to approximately 50% at 50 years of age.[26] In Japan, it is also less than 20% under 20 years, but increases rapidly to the plateau of 80% over the age of 40.[27] In Korea, the prevalence rate is the highest; it has already reached 50% at 5 years of age and is 90% in asymptomatic adults over the age of 20.[28]

The discovery of the presence of helicobacter species DNA in liver material from patients with liver disease has led to the challenging hypothesis that these bacteria may play a role in the evolution of hepatic lesions, from chronic viral hepatitis to cirrhosis and hepatocellular carcinoma (HCC).[29] From an epidemiological aspect, several studies have evidenced a high seroprevalence of *H. pylori* among cirrhotic subjects.[9,10,30,31] Despite the availability of these data worldwide, no conclusive data is available about Egyptian patients despite the high prevalence of HCV in Egypt where the predominant genotype is type 4.

In the present study, the *H. pylori* infection was investigated among patients with HCV with chronic active hepatitis and cirrhosis with different staging. The results revealed that this bacterial infection was significantly higher in the patient groups compared with the healthy control group. Moreover, the *H. pylori* infection was found to increase with the increase in both Child and Meld scores. These results reflect high prevalence of *H. pylori* infection, and this may explain the frequent occurrence of gastro-duodenal ulcer in cirrhotic patients.[10] Furthermore, Ponzetto *et al.*[32] proposed that *H. pylori* is implicated in the pathogenesis and progression of cirrhosis, particularly in HCV-infected individuals, and the involvement of *Helicobacter* spp in HCC seems highly possible.

Chronic hepatitis is an inflammatory disease, and each inflammatory process is characterized by increased levels of pro-inflammatory cytokines such as interleukins 1, 6 (IL-1, IL- 6), tumor necrosis factor (TNF) and by the presence of lympho-mono cellular infiltrate and lymphoid follicle formation.[33] Viruses such as HCV are only capable of limited inflammation, due to shedding of IL-1 receptor in circulation, thereby limiting the possibility of IL-1 binding to cellular receptors.[34] Helicobacters, on the other hand, are strong inducers of the inflammatory cascade;[35] infection with them could lead to the accumulation of extraordinary number of lymphocytes and polymorphonuclear cells in the infected tissue.[36] It has been shown that several *Helicobacter* spp could also secrete a liver-specific toxin that causes hepatocyte necrosis in cell culture and might therefore be involved in damaging liver parenchyma *in vivo* (Meyer-ter-Vehn *et al.*[37])

Hepatitis C virus (HCV) is the major agent in non-A non-B hepatitis with serious complications ranging from chronic inflammatory disease to hepatic cirrhosis and end-stage liver failure or hepatocellular carcinoma. Egypt has unusually high prevalence of hepatitis C, resulting in high morbidity and mortality from liver disease. Approximately 20% of blood donors are seropositive for HCV antibodies.[38] Schistosomiasis is another hepatotropic infection that is a major burden on the Egyptian patient population, particularly in rural societies.[39,40] Coinfection with *Schistosoma mansoni* were repeatedly shown to augment pathogenesis induced by HBV and HCV hepatitis.[41] Coinfection with HCV accelerate advancement of liver disease to chronicity of HCV infection, cirrhosis and hepatocellular carcinoma and high incidence of viral persistence.[42] It was generally believed that following acquisition of *H. pylori,* and in the absence of treatment, infection would persist throughout life. However, based on seroepidemiological studies in adults and children from both developing and developed countries, it appears that the spontaneous elimination of *H. pylori* infection may occur.[43] The mode of transmission of *H. pylori* is not definitively known; however, epidemiological studies suggest person-to-person transmission, by either fecal-oral or oral-oral route, to be the major mechanism.[44] In developing countries, there is evidence for both food-borne transmission and waterborne transmission of *H. pylori.*[45] The case for person-to-person transmission is supported by observations that factors such as lower socioeconomic status, lower levels of education, poorer hygiene and sanitation, and household crowding are associated with a higher prevalence of *H. pylori* infection,[46] and these may explain the coinfections as detected by the current study.

In our HCV patients, the concurrent schistosoma infection was documented largely in *H. pylori*–positive patients versus those who were free of schistosoma infection (*P* = 0.01). Concurrent *S. mansoni* infection with *H. pylori* is associated with reduced prevalence of gastric atrophy, a precancerous condition. It was demonstrated that concurrent *S. mansoni* infection may modify the inflammatory response to gastric *H. pylori* infection, by the reduction of oxyradical-induced DNA damage, apoptosis, cellular proliferation activity and increase in antioxidant production.[47] Recently, it was found that *Schistosoma mansoni* secretes a chemokine-binding protein with prominent anti-inflammatory activity,[48] and this may explain that the concurrent parasitic infection might alter the immune response to *H. pylori* infection. The Th2-like response stimulated by helminth infections might modulate the Th1-like immune response induced by *H. pylori* infection despite chronic inflammation leading to a substantial reduction in mRNA for cytokines associated with a gastric inflammatory response of Th1 cells.[49]

It seems clear that sex being male, age above 40 years and positive schistosoma infection are risk factors for increase in the severity of liver disease as revealed by Meld score in *H. pylori*–positive patients [Table 3]. A comparable result was found by Leone *et al.*[50] Coinfections with schistosomes and *H. pylori* clearly augment the pathogenesis induced by HCV.[41,42,50]

Revealing a relation between liver cirrhosis in HCV-infected patients and the presence of *H. pylori* is the most interesting result of the current study. However, the major drawback is the failure to address the relation between liver cirrhosis and the presence of other enterohepatic *Helicobacter* spp., such as *H. hepaticus, H. bilis and H. pullorum.* Further studies are recommended to explore that relation and to detect the antigenic cross-reactivity between *H. pylori* and other species as reported earlier.[51] These future studies should consider the socioeconomic status of patients and controls as it may have a significant impact.

In conclusion, it may be stated that our results collectively reflect a remarkable increase in the *H. pylori* prevalence with advancing hepatic lesions, and the eradication treatment may prove beneficial in those patients with chronic hepatitis C. Future studies should be prospectively carried out to investigate the effect of *H. pylori* treatment on the cirrhotic status of those patients.
